# Mental Recovery and Running-Related Injuries in Recreational Runners: The Moderating Role of Passion for Running

**DOI:** 10.3390/ijerph17031044

**Published:** 2020-02-06

**Authors:** Jan de Jonge, Yannick A. Balk, Toon W. Taris

**Affiliations:** 1 Human Performance Management Group, Eindhoven University of Technology, P.O. Box 513, 5600 MB Eindhoven, The Netherlands; 2 Department of Social, Health and Organisational Psychology, Utrecht University, P.O. Box 80140, 3508 TC Utrecht, The Netherlands; 3 School of Psychology, Asia Pacific Centre for Work Health and Safety, University of South Australia, P.O. Box 2471, Adelaide 5001, Australia; 4 Department of Work and Organizational Psychology, University of Amsterdam, P.O. Box 19268, 1000 GG Amsterdam, The Netherlands; y.a.balk@uva.nl

**Keywords:** mental recovery, mental detachment, harmonious passion, obsessive passion, running-related injury, recreational running

## Abstract

This pilot study investigates the moderating role of passion for running in the relation between mental recovery from running and running-related injuries (RRIs). We predict that the relation between recovery and injuries is dependent on the level of passion. A cross-sectional survey study was conducted among 246 Dutch recreational runners. Multivariate logistic regression analyses revealed that the negative association between mental recovery after running and RRIs is moderated (i.e., strengthened) by harmonious passion. Put differently, runners who are able to mentally recover well after running were less likely to report RRIs in the case of harmonious passion. Additionally, findings demonstrated that obsessively passionate runners were more likely to report RRIs. Passionate runners may benefit from education programs to help them integrate running more harmoniously with other aspects of life, and to prevent injuries. In addition, they should be educated about the crucial role of appropriate mental recovery from running. Considering mental aspects in running such as mental recovery from running and passion for running seems to be worthwhile to gain a better understanding of the incidence and/or prevalence of RRIs. Future (quasi-experimental) studies should investigate the issues raised here more profoundly.

## 1. Introduction

Running is becoming an increasingly popular activity among participants of recreational sports activities [1]. Although recreational running is in general considered a health-promoting activity with associated benefits such as social participation (e.g., through an increase in running groups and running events [1,2]) and stress reduction [3], running-related injuries (RRIs) occur quite often (e.g., [4]). Incidence and prevalence rates of RRIs reported in the literature are rather high (i.e., up to 80%; e.g., [5,6]). In The Netherlands, injury incidence is 6.1 injuries per 1000 sporting hours, which is about three times higher than the national sports average (i.e., 2.1 injuries per 1000 sporting hours). Specifically, Dutch runners suffer from 710,000 RRIs yearly, of which 220,000 are medically treated [7]. Next to soccer, running is the Dutch sport with the highest number of injuries. Forty percent of RRIs are overtraining/overuse injuries, and approximately one-third concerns a recurrence. Male runners are more often injured but the injury risk is higher among female runners. Runners between 20 and 34 years old are more prone to RRIs, especially female runners. Main injury locations are knees (29%), lower legs (25%), and ankles (17%) [7]. From a societal point of view, RRIs cost Dutch society approximately 10 million euros a year expressed in medical costs and costs due to work-related sickness absence and reduced work productivity [8]. Jungmalm and associates [9] concluded that RRIs can be viewed as recreational runners’ primary enemy, and that the public health gains of keeping runners active and healthy should not be underestimated.

Most researchers agree that the majority of RRIs are sustained as a consequence of structural overtraining/overuse (e.g., [10]), or as a consequence of underrecovery (e.g., [11]). Yet, most existing empirical research on injury prediction and prevention focuses heavily upon the physical aspects of overtraining/overuse and underrecovery (e.g., [12]), and focuses less on their mental aspects, despite the potential role of mental aspects in injury prediction and prevention mentioned in the literature (e.g., [3,10,13,14]). As a result, evidence-based knowledge on the role of mental aspects in RRIs is still in its infancy. For that reason, the aim of the present pilot study is to investigate the role of two particular mental aspects in RRIs, namely mental recovery from running and passion for running. Specifically, using the Dualistic Model of Passion [15,16] as a heuristic framework, this study explores and tests the moderating role of passion in the mental recovery-injury relation.

### 1.1. Mental Recovery and Injuries

Runners are exposed to all kinds of running-related efforts. Next to the plausible and logical physical effort, they have to face mental effort as well [3]. For instance, runners often have to run focused and concentrated during races. Research has shown that it is important to compensate running-related efforts with adequate recovery to prevent RRIs (e.g., [11]). Recovery can generally be defined as a dynamic process of restoration and unwinding in which a person’s functioning and efforts return to their initial levels before the efforts took place [17]. From a physical and physiological perspective, recovery reduces and prevents the accumulation of physical fatigue that leads to poor health. From a psychological perspective, it allows the individual to prepare for current or new efforts [18]. A large body of research has investigated the role of a variety of strategies aimed at promoting physical and physiological recovery from training and race efforts (e.g., [19]). In contrast, studies investigating the role of mental recovery in preventing RRIs are scarce [3,20].

In general, there are different perspectives on recovery. It can be considered as an outcome and a process [21]. Recovery as an outcome refers to a person’s physical, physiological and mental state after a recovery or relaxation period. Recovery as a process refers to the activities and experiences that may lead to a change in functioning and health status. As far as the latter is concerned, several authors have argued that it is not the actual recovery activity which helps recovery (such as going for a walk, watching TV, or taking a nap), but rather the psychological processes and mechanisms behind it (e.g., [17,21,22]). In other words, persons may differ with regard to preferred recovery activities while the underlying psychological processes crucial for recovery may be uniform across persons [22]. These psychological processes and mechanisms are called ‘recovery experiences’ (e.g., [22]). One experience that seems to be very important for recovery to occur is mental detachment from running [3,20]. Mental detachment refers to the personal experience of leaving running behind, to mentally switch off completely, and to forget about running immediately after the run training or race [3,17,20,22]. Mental detachment goes beyond the pure physical absence from running and abstaining from running-related efforts. It implies leaving running behind oneself in psychological terms. We are aware of one recent study among 161 recreational athletes showing that mental detachment was related to reporting less sport injuries [20]. To conclude, a completely recovered runner is not only physically recovered, but is also able to mentally detach from and mentally recover after running. If recovery through effective energy management is successful, health and performance will improve, and runners may report less RRIs accordingly [3].

### 1.2. Passion and Injuries

A mental aspect that has gained more and more attention in sport research is passion [15,16,23]. The Dualistic Model of Passion (DMP; [15,16,24]) defines passion as a strong inclination toward a self-defining activity that people like, value, and consider important, and in which they invest considerable time and energy. The DMP suggests that different individuals can be highly committed to the same extent toward an activity such as running, and yet pursue it in qualitatively different ways. Accordingly, the DMP posits the existence of two specific types of passion: harmonious and obsessive. Harmonious passion (HP) results from an autonomous internalization of an activity into one’s identity, and is characterized by a strong desire to freely engage in the passionate activity [25]. With HP, the passionate activity occupies a meaningful―but not overwhelming―place in one’s life and remains in harmony with other aspects of a person’s life [26]. HP is assumed to lead to flexible persistence: one is in full control of the passionate activity, so that when conditions become harmful, involvement in the activity should decline or even stop [27]. The second type of passion, obsessive passion (OP), also refers to a strong desire to engage in the passionate activity [25]. However, OP overwhelms one’s attention, and is postulated to result from an overcontrolled internalization of an activity into one’s identity. OP is also assumed to lead to rigid persistence: one comes to be fully controlled by the passionate activity at the expense of other activities [25,27]. OP leads the person to value the passionate activity over and above all other important activities. This often leads to conflicts either between the passionate activity and other activities, or with one’s partner and relatives [26]. Empirical findings have been consistent with this conceptualization of passion (e.g., [16,24]). Where both types of passion predict similar commitment to an activity and are part of someone’s identity, they have been found to be differentially associated with various outcomes (e.g., [15,16,24]). There is considerable evidence that HP is positively related to psychological outcomes (e.g., positive affect, flow, self-esteem), whereas OP is either unrelated or negatively related to these (e.g., [16,28,29]). In addition, HP has been shown to be positively associated, whereas OP is negatively associated, with experiences of conflict between one’s passion and other life domains [15,30,31]. With regard to performance as an outcome, both types of passion seem to be important. However, OP may at times lead to higher performance levels than HP [16,25]. Furthermore, research on the DMP lends support for the model in sports and sport-related injuries as well. For instance, a study among 80 student dancers showed that harmoniously passionate dancers reported less acute injuries [27]. In addition, OP was associated with prolonged suffering from chronic injuries as well as more rigid involvement in dance activities when injured, whereas HP was unrelated to chronic injuries. Another study of Vallerand and colleagues [15] found that cyclists with OP were still cycling in winter on icy roads, and thus engaged in risky (i.e., injury-promoting) activities while they may be better abstain from such activities. Similar findings regarding the OP-risky behavior relation were also found in a sample of swing dancers [16] and in a study with professional dancers [32]. Finally, in their study of 170 competitive runners, Stephan and his team [33] found that OP was positively associated with perceived susceptibility to sport-related injury.

### 1.3. Mental Recovery, Passion, and Injuries

The current pilot study investigates the moderating role of passion for running in the relation between mental recovery and RRIs in a sample of recreational runners. First, in the case of HP, runners feel engaged with running but remain in harmony with other important activities of life. They are in full control of the passionate activity and are able to stop it whenever necessary. This implies that runners with HP are able to cease running activities at any time, are able to engage in recovery from running, mentally detach from running after a run, and feeling mentally recovered at the end. So, we expect that mental detachment from running and mental recovery after running will be negatively related to RRIs and that these relations are moderated (i.e., strengthened) by HP (Hypothesis 1). Put differently, harmoniously passionate runners who are able to mentally detach from running and/or recover well after running are less likely to report RRIs. Second, runners with OP have an uncontrollable urge to engage in running, and they highly value it over all other important activities of life. They are fully controlled by the passionate activity (rather than that they are in full control of this activity, as in HP) and will persist in running despite the body and mind signals that recovery is necessary. Thus, obsessively passionate runners will disregard their need for recovery and, hence, will be less able to mentally detach from running as well as to mentally recover after running. Consequently, they may negate minor RRIs and overtrain/overuse, or underrecover, themselves, leading to more serious RRIs in the long run (cf. [10,11]). We expect that mental detachment from running and mental recovery after running will be negatively related to RRIs, and that these relations are moderated (i.e., buffered) by OP in such a way that the associations will be less negative (Hypothesis 2). In other words, the association between (1) mental detachment from running and mental recovery after running and (2) RRIs will be weaker in the case of obsessively passionate runners.

## 2. Materials and Methods

### 2.1. Study Design, Data, and Procedure

A cross-sectional survey study was conducted in the Summer of 2017. Recreational runners were recruited via all Dutch running associations (N = 371) that were mentioned on the website of the Dutch Athletics Foundation (AU). The AU is the national umbrella organization of all Dutch athletics and running clubs, and closely linked to the International Association of Athletics Federations (IAAF) and European Athletics (EA). Both novice and more experienced runners received a unique, secured link to an online survey, where they had to fill out their email address. All participants gave their informed consent for inclusion before they participated in the study. They received information about the aim of the study and voluntary participation, and were told that their data would be handled confidentially. This pilot study was conducted in accordance with the Declaration of Helsinki and the American Psychological Association, and received institutional approval. Moreover, the Medical Ethics Committee of the University Medical Center Utrecht in the Netherlands has exempted our series of survey research studies in runners from further ethical approval (reference number: NL64342.041.17). Initially 254 recreational runners who ran at least once a week returned the questionnaire. The ultimate sample consisted of 246 runners due to some missing data. More than half of the participants were male (53.7%) and 46.3% was female. Mean age was 47.2 years (SD = 13.4; range 19–77). Average running experience was 14.4 years (SD = 12.0). On average, participants engaged in running activities 2.8 times a week (SD = 1.0). The average running distance was 26.5 kilometers per week (SD = 16.6), whereas the average running time was 3.2 hours per week (SD = 1.8). Overall, the average running speed was 10.1 km/h (SD = 18.8). Forty-two participants (17.1%) ran at least four times a week with an average running distance of 47.6 kilometers per week and an average running speed of 9.3 km/h. Ten people (4.1%) ran at least five times a week with an average running distance of 62.2 kilometers per week and an average running speed of 9.5 km/h. Two-thirds of the runners ran in groups (68.0%), and approximately half of the runners (45.5%) used an individualized training schedule for their training activities. Of all participants, 51.2% self-reported RRIs over the past 12 months, such as knee, Achilles tendon and foot injuries. These training and injury figures were comparable to other Dutch studies among recreational runners (e.g., [3,5,34]).

### 2.2. Variables and Instruments

#### 2.2.1. Mental Recovery

We used two scales for mental recovery reflecting the two different perspectives mentioned earlier; that is, mental detachment from running (‘recovery process’) and mental recovery after running (‘recovery outcome’; cf. [21,22]). Scales are available from the first author upon request.

Mental detachment from running was measured with a slightly adapted scale developed by De Jonge and colleagues [35]. This scale had been used and well-validated in sports before (e.g., [3,36]). Participants were asked if they could mentally switch off from running immediately after a run training or race. The scale was measured with three items, e.g., “I could mentally distance myself from running directly after a run”. Items were scored on a 5-point Likert scale, ranging from 1 (never) to 5 (always). Internal consistency of the scale expressed in Cronbach’s alpha was 0.90.

Mental recovery after running was assessed with an adaptation for running of the well-validated recovery measure developed by Sonnentag and Kruel [37] to running. Participants were asked if they feel mentally recovered a couple of hours after a run training or race. The scale consisted of three items, scored on a 7-point Likert scale, ranging from 1 (totally disagree) to 7 (totally agree). An example item is: “A couple of hours after my running activities, I usually feel recovered mentally”. Cronbach’s alpha was 0.90. The factor structure of mental detachment and mental recovery was investigated with a factor analysis (PAF) with oblimin rotation. This factor analysis resulted in an obvious two-factor solution with all detachment items loading on one factor and all recovery items loading on the other. Eigenvalues were 3.50 and 2.15 respectively, explaining 80.7% of the variance. Pearson zero-order correlation for the two scales was *r* = 0.22 (*p* = 0.001), showing that mental detachment after running was positively but moderately related to mental recovery from running.

#### 2.2.2. Harmonious and Obsessive Passion

Harmonious and obsessive passion were measured by the respective scales developed by Vallerand and colleagues [15,16]. The scales were slightly adapted as the passionate activity used here is ‘running’. Harmonious passion emphasized a strong inclination where the runner feels engaged and has full control over running, and the activity is in harmony with the person’s other activities. An example item is: “Running is well integrated in my life”. As one item of the original scale did not pass psychometric scrutiny, our scale consisted of five items, with an internal consistency (Cronbach’s alpha) of 0.79. Obsessive passion reflected a strong inclination where the runner feels compelled to engage in running, running takes a lot of space, the runner loses control over running, and experiences conflict with other life activities. This scale consisted of six items, for instance: “I have almost an obsessive feeling for running” (Cronbach’s alpha = 0.90). Both scales were scored on a 7-point Likert scale, ranging from 1 (do not agree at all) to 7 (completely agree). We tested the factor structure of both passion scales with a factor analysis (PAF) with oblimin rotation. Results revealed a clear two-factor solution with eigenvalues of 4.92 and 1.41, explaining 63.3% of the variance. All OP items loaded on the first factor and all HP items loaded on the second factor. The two scales were not significantly related to each other (*r* = 0.08, *p* = 0.222).

#### 2.2.3. Running-Related Injuries

Running-related injuries were self-reported by runners, and consisted of a time frame of the past 12 months. Based on a consensus definition [38,39], RRIs were defined as: “injuries, impairments or wounds, whether or not associated with pain, caused by or developed during a running training, that causes a restriction on running (in terms of duration, speed, frequency, distance, or intensity) or stoppage of running for at least seven days or three consecutive scheduled training sessions”. In line with other large-scale research studies in RRIs (e.g., [6,34]), we assessed RRIs by means of a well-validated single question with a dichotomous response scale (0 = no; 1 = yes): “In the past 12 months, have you suffered one (or more) sport injuries following the above definition as a result of your running?”. Injuries were overall injuries; no difference was made in acute injuries or overtraining/overuse injuries.

#### 2.2.4. Control Variables

We controlled for gender (0 = female; 1 = male), age (years), use of an individualized training schedule (0 = no; 1 = yes), running distance per week (kilometers), and running time per week (hours). Past studies have shown that these characteristics could have a significant influence on runners’ injuries (e.g., [3,5,34]). In addition, a recent meta-analysis showed that the remaining running-related characteristics are less relevant as control variables [40].

### 2.3. Statistical Analysis

Firstly, means, standard deviations, and Pearson zero-order correlations were calculated using IBM SPSS Statistics 25 (SPSS Inc., Chicago, IL, USA). Secondly, multivariate logistic regression analyses were used to determine the associations between mental detachment, mental recovery, passion, and RRIs. Multivariate odds ratios (ORs) and 95% confidence intervals (CIs) were derived from the logistic regression models. In all analyses, gender, age, training schedule use, running distance and running time were controlled for. Postulated moderating effects of passion (i.e., HP and OP) with recovery (i.e., mental detachment and mental recovery) were tested by adding multiplicative interaction terms (recovery × passion) of standardized recovery and passion scales into the regression model. Since we expected differential effects for the two passion scales, we performed two regression analyses accordingly: one for HP and one for OP. Nagelkerke *R*^2^ was used as an approximation of the explained variance of the logistic regression model.

## 3. Results

Means (M), standard deviations (SD), and Pearson zero-order correlations for the different variables are displayed in Table 1. A first inspection of the Pearson zero-order correlations shows that our control variables were moderately but significantly related to several predictor variables and outcome variables. For instance, age was significantly related to mental detachment from running (*r* = 0.24, *p* = 0.000), mental recovery after running (*r* = 0.21, *p* = 0.001), HP (*r* = 0.21, *p* = 0.001), and RRIs (*r* = −0.13, *p* = 0.046). Next, gender was significantly associated with both running distance (*r* = 0.23, *p* = 0.000) and running time (*r* = 0.20, *p* = 0.002), while age was significantly related to running time (*r* = 0.19, *p* = 0.003). Interestingly, mental detachment from running was significantly and negatively linked to running distance (*r* = −0.24, *p* = 0.000) and running time (*r* = −0.21, *p* = 0.001) as well. Finally, both HP (*r* = −0.15, *p* = 0.022) and OP (*r* = 0.14, *p* = 0.026) as well as the interaction between mental recovery after running and HP (*r* = −0.13, *p* = 0.048) were moderately associated with RRIs.

Table 2 depicts the logistic regression results for RRIs, which showed support for an interaction model in the case of HP and, hence, a moderating effect of mental recovery after running and HP. Specifically, the negative association between mental recovery after running and RRIs is moderated (i.e., strengthened) by HP. Put differently, harmoniously passionate runners who are able to mentally recover well after running were 0.72 times (or 28%) less likely to report RRIs (OR = 0.72; 95% CI = 0.54–0.96; *p* = 0.031). However, findings did not show an interaction effect of mental detachment from running and HP, and did not show direct negative associations between mental recovery after running, mental detachment from running and RRIs as well. Overall, the predictor variables were able to explain 10.4% of the variance in RRIs. At the end, the classification accuracy shows that this prediction was correct 62.2% of the time. With respect to OP, logistic regression results showed a main effect-only model rather than an interaction model. Findings demonstrated that obsessively passionate runners were 1.36 times (or 36%) more likely to report RRIs (OR = 1.36; 95% CI = 1.03–1.85; *p* = 0.047) than others. Again, findings did not show direct negative associations between mental recovery after running, mental detachment from running and RRIs. Nagelkerke *R*^2^ shows that the predictor variables together were able to explain 7.9% of the variance in RRIs. Finally, the classification accuracy shows that this prediction was correct 59.2% of the time.

## 4. Discussion

The general purpose of this pilot study was to investigate the moderating role of passion for running in the relation between mental detachment from running, mental recovery after running and running-related injuries (RRIs). Based upon scientific literature and preliminary evidence, we formulated and tested two hypotheses accordingly. First, we hypothesized that both mental recovery components (i.e., mental detachment from running and mental recovery after running) will be negatively related to RRIs, and that this relation is moderated (i.e., strengthened) by HP. Findings do show the expected interaction effect between mental recovery after running and HP in the prediction of RRIs. In other words, there is only a negative association between mental recovery after running and RRIs in the case of being a harmoniously passionate runner. This supports Hypothesis 1. However, findings did not show the expected interaction effect of mental detachment from running and HP in the prediction of RRIs, which is not in support of Hypothesis 1. Second, we hypothesized that both mental recovery components will be negatively related to RRIs, and that this relation is moderated (i.e., buffered) by OP in such a way that the associations will be weaker. Results do not show the proposed interaction effect between mental recovery and OP in the prediction of RRIs. Instead, they only demonstrate a main effect of OP in the prediction of RRIs. In other words, obsessively passionate runners are more likely to report RRIs. These findings do not support Hypothesis 2. Finally, the predictor variables were able to explain about 8% to 10% of the variance in RRIs, and the predictions were correct in approximately 60% of the time. Although these effects are not very strong, they are interesting and promising.

### 4.1. Theoretical Implications

Findings with regard to the two mental recovery components (i.e., mental detachment from running and mental recovery after running) and RRIs are interesting. Although mental recovery after running is in general considered as being beneficial for injury prevention (e.g., [3,20]), this study shows that this may particularly be the case with harmoniously passionate runners. HP is characterized by a more flexible psychological state that should lead the runner to focus and to concentrate better, to experience less pressure, and to relax better accordingly (cf. [15,16]). In addition, mental recovery research showed that mental recovery potential is highest in cases where the need for recovery is intrinsically motivated [41]. HP could be such an intrinsic motivator.

The present study extends the work of Balk and associates [20] who showed that mental detachment from running was related to athletes’ report of less injuries. In our study, however, it is not mental detachment from running but mental recovery after running in harmoniously passionate runners which seems to be negatively associated with RRIs. As both recovery measures are self-report instruments, an explanation for this finding is that recovery *outcome* measures seem to be more sensitive and concrete mental recovery measures than recovery *process* measures [21]. While the recovery outcome is related to the recovery process, it is the concrete mental recovery state directly after running which matters most for harmoniously passionate runners in the prediction of their RRIs. Moreover, if one moves beyond self-report ratings in the direction of more objective data (e.g., psycho-physiological data), disentangling the recovery process from the recovery outcome will be more difficult, or even not possible at all [21]. To conclude, this study shows that, for harmoniously passionate runners, mental recovery after running as an outcome of a successful recovery process is more important than mental detachment from running as part of the process itself to predict less RRIs.

The findings for passion for running are consistent with previous research on the concept of passion and the Dualistic Model of Passion [15,16]. The two types of passion demonstrate the way running has been internalized into a runner’s identity: HP in which the person controls the activity, and OP where the activity controls the person [15]. In line with earlier passion research [16,27,33], it might be that harmoniously passionate runners are being able to detect early warning signals related to injuries and to adopt precautionary behavior such as taking a mental break in time. Conversely, obsessively passionate runners cannot stop running even when positive returns are no longer forthcoming and running has become harmful to them [15]. The non-existence of interaction effects between mental detachment, mental recovery and OP could be explained by the fact that obsessively passionate runners are not capable of detecting early warning signals related to injuries as well as adopting precautionary behavior such as taking a mental break in time. In other words, they will disregard their need for recovery and, hence, will not be able to mentally detach from running as well as to mentally recover after running. Thus, while such rigid persistence to running may initially lead to benefits such as improved performance, it may also come at personal costs such as RRIs in the end.

Finally, since obsessive passion is considered to be one of the key predictors for exercise addiction [42], our results are also consistent with research on exercise and running addiction (e.g., [42,43,44,45]). Exercise addiction can be defined based on the same criteria used to define other addictive behaviors including tolerance, withdrawal, lack of control, time, reduction in other rewarding activities, and continuance despite negative outcomes [46]. Of all the types of sports studied, endurance sports such as long-distance running are those showing the greatest risk of addiction [42,43]. For example, runners who find they need to run more to experience the same positive neuropsychological effects (e.g., runners high), experience irritability or even depression when unable to run, find that running time interferes with responsibilities in other domains (e.g., work or family), or exercise despite RRIs may have an addictive-like relation with exercise [47]. In a literature review of 25 empirical studies, Nogueira and associates [42] concluded that excessive practice may indeed cause the appearance of addictive behaviors and serious health problems. A recent study of Martin and her team [47] has highlighted the fact that endurance runners with high levels of exercise addiction pressed on in spite of the negative consequences brought about by not running, because the compensation they derive is greater than any rewards from not doing so.

### 4.2. Limitations and Future Research Directions

Besides its valuable insights, this study has several limitations. A first limitation concerns its cross-sectional design which does not permit any causal conclusions for the variables under study. However, this was due to the pilot character of this study. A two-wave cross-lagged panel study by Carbonneau and associates [48] in a non-sports sample showed that passion leads to changes in outcomes, but not the other way around. Further research using longitudinal study designs is needed to replicate and corroborate current findings (cf. [3]). Such studies would also contribute to the understanding of sports-related, social and psychological factors that promote or hinder the development of one type of passion over the other [27]. A second limitation is that common method variance due to using self-report data may have played a role, although recent research studies have shown that this influence is not as strong as sometimes believed (e.g., [49]). This risk was minimized by measuring our self-report scales as objective as possible (‘facts’) with clear instructions to fill out, accompanied with concrete and different response rates as well as profound tests on validity and reliability. The risk was further reduced by assessing the outcome measure with a different response format and anchors compared to the predictor variables, as suggested by Podsakoff, MacKenzie, and Podsakoff [50]. A third limitation is that self-reported RRIs were used. This implies that the runners had to judge the injuries themselves, without a formal diagnosis from a medical practitioner. This is partly solved by providing the runners with a clear consensus definition of RRIs as well as using the same survey question as used in other, large-scale, empirical research (e.g., [6,34]). Furthermore, the quality of RRIs was not taken into account. For instance, RRIs due to overuse or overtraining might be qualitatively different in their genesis than RRIs due to trauma. Similarly, the seriousness of RRIs might vary greatly and could have an impact on recovery schemes. It is also recommended to add more formal and comprehensive diagnostic information of RRIs by practitioners, which could enhance a study’s validity in future research. Fourth, although we found direct associations between passion and RRIs, we do not know if some runners in our sample were physically predisposed to RRIs. A physical screening program at forehand would be recommended in this respect. Fifth, our logistic regression models have been adjusted for various control variables. Nevertheless, the question remains which other control variables such as participation in competition or extent performance level are associated with HP and OP. Future research might therefore consider assessing similar questions in different groups of runners. Sixth, current findings are likely to be valid for all types of recreational runners. However, it is plausible that the associations are underestimated due to the absence of elite runners (i.e., restriction of range effect). Finally, given the current sample of recreational runners, its sample size and pilot character, future research is needed whether or not the current results will hold in other samples of recreational and elite athletes as well. An example of such research is a randomized controlled trial with a 12-month follow-up [3]. After completing a web-based baseline survey, 425 half and full marathon runners were randomly assigned to either an intervention group or a control group. Participants of the intervention group obtained access to an online injury prevention programme, consisting of a running-related smartphone application and activity trackers. The smartphone application provided the participants of the intervention group with information on how to prevent overtraining/overuse and RRIs with special attention to mental aspects such as mental recovery, passion and mental fatigue. Due to a wait list control group design, participants in the control group got access to the application and related preventive information after the first follow-up measurement as well. Data collection and analysis is in progress, and will be published elsewhere (cf. [3]).

### 4.3. Practical Implications

The present study demonstrates the important role of passion in the relation between mental recovery and RRIs. Because many runners are devoted to and passionate about their sports, it is important to help them understand that there are two different types of passion: harmonious passion and obsessive passion. HP entails control over running and a harmonious co-existence of running with other activities in life, such as adequate mental and physical recovery. In contrast, OP entails little or even lack of control over running, rigid persistence, and conflict with other activities in a runner’s life. So, HP seems to be a more desirable type of passion than OP in the case of RRIs, and runners should be encouraged to develop a more harmonious passion in this respect (cf. [26,27]). However, this does not mean that OP is negative. It may not lead to outcomes as adaptive as those derived from HP, but OP is still more adaptive than being amotivated [15]. For instance, benefits from OP are reflected by the immediate positive consequences associated with increased performance (e.g., [16,25]). Further, OP may lead to long-term commitment and persistence in running, despite its potential countereffects on RRIs. Passionate runners may benefit from education programs in order to help them integrate running more harmoniously with other aspects of life. In addition, they should be educated about the crucial role of appropriate recovery from and after running. Moreover, run coaches and trainers should be aware of the two types of passion as well, and how they characterize different ways running has been internalized into a runner’s identity. Periodized training schemes and smartphone applications could then be adapted to the individual runner (cf. [3]), and ideally should take into account how to take mental breaks next to regular physical breaks (cf. [20]). Our study shows that this is particularly relevant for obsessively passionate runners.

## 5. Conclusions

This pilot study in recreational runners suggests that particularly the combination of harmonious passion for running and mental recovery after running is important to predict and prevent RRIs. Moreover, it suggests that obsessive passion for running is a mental risk factor for RRIs itself. So, considering mental aspects in running seems to be valuable to gain a better understanding of the incidence and/or prevalence of RRIs. Preventing and/or reducing RRIs will facilitate runners to remain active, which in turn may contribute to their health, vitality and sustainable performance―not only in sports but also in work and private life activities [51]. This can reduce medical costs and costs due to absence from work as well. Further research on the issues raised here would be rather promising.

## Figures and Tables

**Table 1:** Descriptive statistics and Pearson zero-order correlations among study variables (*n* = 246).

Variables	M	SD	1	2	3	4	5	6	7	8	9	10	11	12	13
1. Gender (0 = female; 1 = male)	0.54 #	0.50													
2. Age (years)	47.15	13.41	0.26 **												
3. Training schedule (0 = no; 1 = yes)	0.46 #	0.50	−0.07	−0.01											
4. Running distance (km)	26.53	16.56	0.23 **	0.08	0.14 *										
5. Running time (hours)	3.20	1.82	0.20 **	0.19 **	0.08	0.69 **									
6. Mental detachment from running	2.84	1.19	0.01	0.24 **	−0.11	−0.24 **	−0.21 **								
7. Mental recovery after running	5.67	1.16	0.11	0.21 **	0.04	0.02	0.05	0.22 **							
8. Harmonious passion (HP)	2.60	1.35	−0.01	0.21 **	−0.10	−0.34 **	−0.26 **	0.32 **	0.17 **						
9. Obsessive passion (OP)	3.41	1.50	−0.05	−0.10	0.13 *	0.37 **	0.29 **	−0.43 **	−0.17 **	0.08					
10. Mental detachment × HP	0.32	1.10	0.06	−0.14 *	0.12	0.23 **	0.09	−0.06	−0.04	−0.25 **	0.05				
11. Mental recovery × HP	0.17	1.00	0.05	−0.01	0.07	0.11	0.09	−0.05	−0.17 **	−0.09	0.05	0.13 *			
12. Mental detachment × OP	−0.43	0.99	0.05	0.11	−0.06	−0.17 **	−0.09	−0.07	0.02	0.05	−0.04	−0.43 **	−0.11		
13. Mental recovery × OP	−0.17	0.96	0.07	0.06	−0.04	0.04	0.02	0.02	0.29 **	0.05	0.05	−0.12	−0.55 **	0.20 **	
14. RRIs (0 = no; 1 = yes)	0.51 #	0.50	0.07	−0.13^*^	−0.07	0.05	0.10	−0.11	−0.04	−0.15 *	0.14 *	0.05	−0.13 *	−0.06	0.04

* Significant at *p* < 0.05; ** significant at *p* < 0.01 (two-tailed); # these are dichotomous variables, their means can thus be interpreted as a percentage.

**Table 2:** Logistic regression models of running-related injuries with detachment, recovery and passion as predictor variables (*n* = 246).

	Running-Related Injuries					
	Harmonious Passion			Obsessive Passion		
	B	SE	OR (95% CI)	B	SE	OR (95% CI)
Control variables						
Gender	0.46	0.29	1.59 (0.90, 2.81)	0.49	0.28	1.63 (0.92, 2.88)
Age	−0.02	0.01	0.98 * (0.96, 0.99)	−0.03	0.01	0.96 * (0.94, 0.98)
Training schedule use	−0.40	0.28	0.67 (0.39, 1.15)	−0.37	0.27	0.69 (0.41, 1.17)
Running distance	−0.01	0.01	0.99 (0.96, 1.02)	−0.01	0.01	0.99 (0.96, 1.02)
Running time	0.17	0.13	1.18 (0.92, 1.51)	0.16	0.12	1.17 (0.92, 1.48)
Predictor variables						
Mental detachment from running	−0.03	0.15	0.97 (0.72, 1.32)	0.02	0.16	1.02 (0.75, 1.39)
Mental recovery after running	−0.02	0.14	0.98 (0.74, 1.30)	0.03	0.14	1.02 (0.78, 1.35)
Harmonious passion (HP)	−0.31	0.17	0.73 (0.53, 1.02)			
Obsessive passion (OP)				0.32	0.15	1.36 * (1.03, 1.85)
Moderating variables						
Mental detachment × Passion (HP)	0.02	0.16	1.02 (0.81, 1.36)			
Mental recovery × Passion (HP)	−0.32	0.14	0.72 * (0.54, 0.96)			
Model test	χ^2^ = 19.37, *df* = 10, *p* = 0.036			χ^2^ = 15.89, *df* = 8, *p* = 0.044		
Nagelkerke *R*^2^	10.4%			7.9%		
Classification accuracy	62.2%			59.2%		

* Significant at *p* < 0.05 (two-tailed).
